# For Cholera Infantum

**Published:** 1887-01

**Authors:** 


FOR CHOLERA INFANTUM.—

| | | |
|---|---|---|
| ℞ | Argenti nitrat., - - - - | gr. j |
| | Acid nit. dilt., - - - | gtt. viij |
| | Tr. opii., - - - - - | gtt. viij |
| | Mucilag. acaciæ, - - - | fl℥ ss |
| | Syrupi, - - - - - | fl℥ ss |
| | Aquæ, - - - - - | fl℥ j |

